# The City of London (Chest) Hospital

**Published:** 1920-12-25

**Authors:** 


### The City of London (Chest) Hospital.

At the City of London (Chest) Hospital, Victoria Park, E. 2, increased costs bring the expenditure this year to £33,000, and make the financial outlook serious. The hospital's work is of supreme importance, owing to the increased ravages of consumption, of which the heavy death-rate throughout the kingdom is a dreadful evidence. The deficiency in accommodation generally for the treatment of the disease makes the hospital's efforts towards shouldering this burden in the East End of enormous value. To enable it to continue the work unimpaired, with its beds, numbering 175, and extensive out-patient department, further help is urgently needed. All patients able to do so now contribute. Donations will be gladly received at the hospital or by Barclays Bank, Ltd., 54 Lombard Street, E.C. 3.

